# Lapatinib-capecitabine versus capecitabine alone as radiosensitizers in RAS wild-type resectable rectal cancer, an adaptive randomized phase II trial (LaRRC trial): study protocol for a randomized controlled trial

**DOI:** 10.1186/s13063-016-1583-y

**Published:** 2016-09-21

**Authors:** Nuno Sousa, Olga Sousa, Lúcio Lara Santos, Rui Henrique, Manuel R. Teixeira, Mário Dinis-Ribeiro, Armando Teixeira-Pinto

**Affiliations:** 1 Medical Oncology Department, IPO Porto FG, EPE, Rua António Bernardino de Almeida, 4200-072 Porto, Portugal; 2 Radioncology Department, IPO Porto FG, EPE, Rua António Bernardino de Almeida, 4200-072 Porto, Portugal; 3 Surgical Oncology Department, IPO Porto FG, EPE, Rua António Bernardino de Almeida, 4200-072 Porto, Portugal; 4 Department of Pathology, IPO Porto FG, EPE, Rua António Bernardino de Almeida, 4200-072 Porto, Portugal; 5 Department of Pathology and Molecular Immunology, Abel Salazar Institute of Biomedical Sciences – University of Porto, Rua de Jorge Viterbo Ferreira, no. 228, 4050-313 Porto, Portugal; 6 Genetics Department, IPO Porto FG, EPE, Rua António Bernardino de Almeida, 4200-072 Porto, Portugal; 7 Biomedical Sciences Institute Abel Salazar (ICBAS), University of Porto, Porto, Portugal; 8 Gastroenterology Department, IPO Porto FG, EPE, Rua António Bernardino de Almeida, 4200-072 Porto, Portugal; 9 CINTESIS - Center for Health Technology and Services Research, Centro de Investigação Médica, Faculdade de Medicina da Universidade do Porto, Rua Dr. Plácido da Costa, s/n, 4200-450 Porto, Portugal; 10 Screening and Test Evaluation Program (STEP), Sydney School of Public Health, The University of Sydney, Sydney, NSW 2006 Australia

**Keywords:** Rectal neoplasms, Radiotherapy, Capecitabine (capecitabine [supplementary concept]), Lapatinib (lapatinib [supplementary concept]), Neoadjuvant therapy, Clinical trial, Phase II, Randomized adaptive trial, Bayesian adaptive randomization

## Abstract

**Background:**

Preoperative radiochemotherapy followed by surgical removal of the rectum with total mesorectum excision is the preferred treatment option for stages II and III rectal cancer. However, development of metastatic disease is the main cause of death for these patients with 5-year disease-free survival rates of 56 %. Anti-epidermal growth factor receptor (EGFR) targeted therapy is effective in metastatic rectal cancer, and human epidermal growth factor receptor 2 (HER-2) signaling may mediate resistance to EGFR inhibitors. Moreover, preclinical data support a synergistic effect of EGFR inhibition with radiation therapy.

**Methods/design:**

This Bayesian phase II trial with adaptive randomization was designed to assess the efficacy of adding lapatinib, a dual inhibitor of EGFR and HER-2, to standard radiochemotherapy with capecitabine in stages II and III rectal cancer.

**Discussion:**

The results of this trial will provide evidence of the feasibility and efficacy of the combination of lapatinib-capecitabine as radiosensitizers and explore potential predictive biomarkers for response to this novel neoadjuvant approach to resectable rectal cancer.

**Trial registration:**

EudraCT 2013-001203-36. Registered on 13 December 2013.

## Background

Colorectal cancer is the third most incident cancer and the second most common cause of cancer death in Europe [1]. Cancer of the rectum accounts for a third of colorectal cancer cases [2] with 5-year survival rates ranging from 28–55 % in men and 30–64 % in women [3].

Current treatment options for patients with rectal cancer include surgery, radiation therapy, chemotherapy, and targeted systemic therapy. The choice of therapy depends on disease stage, overall health status, and patient preferences. For localized disease, surgical removal of the rectum with total mesorectum excision is the surgical technique of choice, with an estimated overall survival of 86 % and a local recurrence rate of 9 % at 2 years [4]. However, the use of radiotherapy before surgical resection decreases local recurrence rate and impacts overall survival [5]. Moreover, the addition of fluoropyrimidine-based chemotherapy to preoperative radiotherapy further improves local control and pathologic response rates [6]. In patients treated with preoperative radiochemotherapy, whenever downstaging occurs, the risk of death due to cancer is significantly reduced [7]. Despite these therapeutic improvements, patients with localized rectal cancer have 5-year disease-free and overall survival rates of about 59 % and 67 %, respectively [8, 9]. For these patients, development of metastatic disease is the main cause of death.

Capecitabine is a non-cytotoxic fluoropyrimidine carbamate, which functions as an orally administered precursor of the cytotoxic agent 5-fluorouracil. Capecitabine is active as a single agent in colorectal cancer, and it is approved for the adjuvant and palliative treatment of patients with colorectal cancer [10]. Its value as a radiosensitizer in rectal cancer has been established by several phase II trials, with pathologic response rates that range from 7–38 % [11–15]. In a phase III non-inferiority trial, radiochemotherapy with capecitabine was shown to be non-inferior to 5-fluorouracil radiochemotherapy, with survival rates of 76 % at 5 years [16]. Taken together, these data and the more comfortable administration regimen of capecitabine compared to infusional 5-fluorouracil make it the preferential agent for the neoadjuvant treatment of patients with rectal cancer.

Colorectal cancer frequently overexpresses epidermal growth factor receptor (EGFR) [17]. Ligand binding to the extracellular portion of the receptor promotes homodimerization or heterodimerization and subsequent activation of the receptor. This leads to increased cell growth, proliferation, and mobility, decreased apoptosis, and stimulation of angiogenesis. Currently available drugs targeting the EGFR include monoclonal antibodies (e.g., cetuximab and panitumumab) and small molecule tyrosine kinase inhibitors (e.g., gefitinib, erlotinib, and lapatinib). In metastatic colorectal cancer, cetuximab and panitumumab were shown to be clinically effective [18–23]. Resistance to anti-EGFR antibodies may be mediated by HER-2, since the addition of anti-HER-2 targeting agents to cetuximab is able to overcome this resistance [24, 25]. These data support the development of therapeutic strategies targeting both EGFR and HER-2 in colorectal cancer. Additionally, targeting the EGFR pathway during neoadjuvant radiochemotherapy is associated with an increased number of cells in the G1 phase of the cell cycle (the most radiosensitive phase) and increased apoptosis, and prevents repair of DNA damage induced by radiation exposure, thus suggesting a possible synergistic effect of EGFR inhibition with radiation therapy [26, 27]. This strategy has been explored with cetuximab and gefitinib in combination with fluoropyrimidine-based radiochemotherapy in the phase I/II setting with disappointing results [28–30]. However, all these studies were conducted before the predictive role of RAS mutations in anti-EGFR treated patients was recognized; therefore, they may have been underpowered to detect a clinically relevant improvement for this therapeutic strategy.

Lapatinib is a small molecule dual tyrosine kinase inhibitor targeting both EGFR and HER-2. Preclinical studies have shown that lapatinib is active in colorectal cancer cell lines [31, 32], and its radiosensitizing properties have also been demonstrated in preclinical models [33, 34]. In treating metastatic colorectal cancer, lapatinib has been shown to be well tolerated, both as a single agent and in combination with chemotherapy, but with limited activity [35–38]. However, no population enrichment strategy based on tumor characteristics was implemented, and these trials may have been underpowered to identify any clinically relevant activity. Considering the clinical value of anti-EGFR therapy in RAS wild-type colorectal cancer [19–23, 39] and the theoretical advantage of anti-EGFR treatment during radiotherapy [26, 27] together with the data suggesting that dual inhibition of EGFR and HER-2 may be synergistic [31, 32], one can hypothesize that lapatinib is an ideal candidate for optimizing the neoadjuvant treatment of rectal cancer.

A randomized phase II trial with an adaptive Bayesian randomization design is proposed to assess the efficacy and safety of the tyrosine kinase inhibitor lapatinib combined with the fluoropyrimidine analog capecitabine as radiosensitizers in patients with clinical stage II/III resectable rectal cancer without activating RAS mutations.

## Methods/design

This single-center phase II trial is designed with an adaptive Bayesian randomization, which will allocate eligible patients to either lapatinib-capecitabine (arm A) or capecitabine (arm B) during standard radiation treatment for rectal cancer. The first 20 patients will be allocated at a 1:1 ratio. From that time point onward the randomization of a patient to either arm will be weighted by the updated estimate of the success probability in each arm, hence giving patients a higher chance of being allocated to the current most efficacious arm of the trial. The trial is designed to be stopped earlier if evidence of superiority of one arm over the other, futility, or unacceptable toxicity in arm A is identified.

### Population and setting

The trial will be implemented at Instituto Português de Oncologia do Porto Francisco Gentil, EPE, where patients will be considered eligible for the trial based on the following criteria.

#### Inclusion criteria

Each patient must meet all of the following inclusion criteria to be enrolled in the study:

1. Age of 18 years or above
2. Diagnosis of adenocarcinoma of the rectum with the primary malignant lesion located between the dentate line up to 10 cm of the anal verge by endoscopic examination
3. Absence of activation mutation in KRAS exons 2 (codons 12 and 13), 3 (codons 59 and 61), and 4 (codons 117 and 146) and NRAS exons 2 (codons 12 and 13), 3 (codons 59 and 61), and 4 (codons 117 and 146)
4. Clinical stage II or III disease, according to American Joint Committee on Cancer Staging classification, 7th edition [40]. For clinical staging, the following procedures must have been conducted within 4 weeks of treatment allocation:
  - Clinical history and physical examination
  - Endoscopic ultrasound of the primary lesion
  - Thoracic, abdominal, and pelvic computed tomographic (CT) scan
5. Clinically judged to be able to undergo curative resection of the rectal neoplasm despite preoperative radiochemotherapy
6. Clinically judged to be able to undergo pelvic radiation therapy to a total dose of 50.4 Gy
7. Provision of signed informed consent

#### Exclusion criteria

Patients meeting any of the following exclusion criteria are not to be enrolled in the study:

1. Absence of baseline histological sample of the primary tumor
2. Pregnant or lactating women
3. Unwillingness or inability to comply with effective contraception, if the patient is fertile.
4. Impaired renal function defined as creatinine clearance <60 mL/min according to the Cockcroft-Gault formula
5. Impaired hematological function as defined by any of the following on pretreatment evaluation:
  - Hemoglobin concentration <10.0 g/dL,
  - Absolute neutrophil count <1500/μL
  - Platelet count <100,000/μL
6. Impaired hepatic function defined by any of the following on pretreatment evaluation:
  - Serum level of aspartate aminotransferase >1.5 × ULN,
  - Serum level of alanine aminotransferase >1.5 × ULN,
  - Serum level of alkaline phosphatase >1.5 × ULN,
  - International normalized ratio >1.5
  - Serum concentration of total bilirubin >1.5 × ULN
7. Symptomatic heart failure or a left ventricular ejection fraction (LVEF) below the institution’s lower limit of normality (LLN) as assessed through equilibrium radionuclide angiography
8. Known intolerance to any of the study drugs.
9. Known dihydropyrimidine dehydrogenase (DPD) deficiency
10. Concurrent treatment with CYP3A4 inducers (as detailed in the following section)
11. Concurrent treatment with CYP3A4 inhibitors (as detailed in the following section)
12. Likely inability to comply with the protocol or cooperate fully with the investigator and site personnel
13. Current enrollment in another clinical trial.

### Trial procedures and study interventions

Recruitment and enrollment for this study will be from the institution’s local practice or referrals from other physicians. All trial-related procedures and interventions will be performed according to the predefined schedule of events (Table 1). Before any study-related procedure, each patient will provide written informed consent, unless that procedure was performed as part of the patient’s standard medical care and is eligible for trial enrollment. Prior to study treatment a screening period, no longer than 28 days, will be considered. During this period, eligibility will be assessed and tumor tissue procured for biomarker analysis. Eligible patients will be randomly allocated to an experimental arm (arm A), which will test the addition of lapatinib to radiochemotherapy with capecitabine or a control arm (arm B), which will consist of standard radiochemotherapy with capecitabine. Patients allocated to arm A will undergo an initial lead-in period of 14 days with lapatinib before radiochemotherapy. At the end of the lead-in period, patients in arm A will have a tumor biopsy performed for biomarker assessment. As lapatinib is a substrate for CYP3A4, inducers and inhibitors of this enzyme are prohibited from screening through discontinuation from study, and a predefined wash-out period prior to random allocation has been set (Table 2). Seven to eight weeks after completion of radiochemotherapy, patients will be surgically treated. For the duration of the trial, no other concurrent anticancer therapy or investigational agents are allowed. During the trial, neither patients nor treating physician will be blinded to treatment interventions.

**Table 1:** Schedule of events

	Screening	Random allocation (^a^)	Treatment period								EOT	Surgery	EOS
			Day -14 (^b^)	Day -7	Day 1 (^c^)	Day 8 ± 3 days	Day 15 ± 3 days	Day 22 ± 3 days	Day 29 ± 3 days	Day 36 ± 3 days	Day 66 ± 3 days	Day 87–94	28 (+7) days after surgery
Informed consent	X												
Inclusion/exclusion criteria	X	X											
Demographics	X												
Medical history	X												
Concomitant medications	X	X		X	X	X	X	X	X	X	X		X
Adverse events				X	X	X	X	X	X	X	X		X
Physical examination	X	X											
Vital signs	X	X		X	X	X	X	X	X	X	X		X
Weight	X	X		X	X	X	X	X	X	X	X		X
Height	X												
ECOG performance status	X	X		X	X	X	X	X	X	X	X		X
Electrocardiogram (12-lead)	X										X		X
MUGA	X										X		X
Hematology	X	X		X	X	X	X	X	X	X	X		X
Serum chemistries	X	X		X	X	X	X	X	X	X	X		X
Hemostasis	X												
Pregnancy test	X										X		X
CT – scan (chest, abdomen, pelvis)	X												
Endoscopic ultrasound	X												
Tumor sampling	X				X(^c^)							X	
Lapatinib (arm A)			1 250 mg/d until the last day of radiotherapy										
Capecitabine (arms A and B)					825 mg/m2 twice daily from first to last day of radiotherapy								
Radiation therapy					50.4 Gy in 28 fractions of 1.8 Gy, 5 days per week								

*EOT* end of treatment visit, *EOS* end of study visit

(^a^) Within 4 weeks of screening visit

(^b^) Not more than 3 days before day -14 in arm A or more than 14 days before day 1 in arm B. In arm B, random allocation visit procedures are valid for day 1 visit if <7 days have elapsed

(^c^) Arm A only: to be performed within a 3-day time window of day 1 of radiation treatment

**Table 2:** CYP3A4 inducers and inhibitors

Drug class	Agent	Wash-out
CYP3A4 inducers		
Antibiotics	All rifamycin agents (e.g., rifampicin, rifabutin, rifapentine)	14 days
Anticonvulsants	Phenytoin, carbamazepine, barbiturates (e.g., phenobarbital)	
Antiretrovirals	Efavirenz, nevirapine	
Glucocorticoids (oral)	Cortisone (>50 mg), hydrocortisone (>40 mg), prednisone (>10 mg), methylprednisone (>8 mg), dexamethasone (>1.5 mg)	Not Applicable
Others	St. John’s wort (*Hypericum perforatum*), modafinil	Not Applicable
CYP3A4 inhibitors		
Antibiotics	Clarithromycin, erythromycin, troleandomycin	7 days
Antifungals	Itraconazole, ketoconazole, fluconazole (>150 mg/d), voriconazole	
Antiretrovirals, protease inhibitors	Delavirdine, nelfinavir, amprenavir, ritonavir, indinavir, saquinavir, lopinivir	
Calcium channel blockers	Verapamil, diltiazem	
Antidepressants	Nefazodone, fluvoxamine	
GI agents	Cimetidine, aprepitant	
Others	Grapefruit, grapefruit juice	
	Amiodarone	6 months
Miscellaneous		
Antacids	Mylanta®, Maalox®, Tums®, Rennie®	1 h before and after dosing
Herbal or dietary supplements	Ginkgo biloba, grape seed, valerian, ginseng, echinacea, evening primrose oil	14 days

#### Lapatinib

Lapatinib, 250-mg tablets, will be self-administered, on an empty stomach (either 1 h before or after a meal), with water, once daily at approximately the same time each day, at a dose of 1250 mg/d. Treatment with lapatinib will be initiated 14 days prior to radiation therapy (lead-in period) and continued for the duration of radiation therapy. Patients will be instructed not to take lapatinib with grapefruit or grapefruit juice. Moreover, patients will be instructed that if they vomit any time after taking a dose, they must not “make it up” with an extra dose, but instead resume dosing with the next scheduled daily dose. Any missed dose will not be replaced; the dosing should resume with the next scheduled daily dose. Patients will be required to return all used, unused, and/or partially used bottles of lapatinib, and the number of remaining tablets will be documented and recorded to assess compliance.

#### Capecitabine

Capecitabine, 150-mg tablets and 500-mg tablets, will be self-administered at a dose of 825 mg/m^2^ (dose rounded to the nearest possible combination of the available tablet formulations) twice daily, within 30 minutes of a meal, with water, for the duration of radiotherapy. Patients will be required to return all used, unused, and/or partially used blister packs of capecitabine at the next study visit. The number of tablets remaining will be documented and recorded to assess compliance.

#### Radiation therapy

Patients will be treated with megavoltage energy to a total treatment dose of 50.4 Gy to the clinical target volume. This treatment will be administered in 1.8-Gy daily fractions, 5 days per week. The treatment technique will be decided by a senior radiotherapist with expertise in rectal cancer treatment. The following techniques will be available: 3D conformal external beam radiation (3D-RT), intensity modulated radiotherapy (IMRT), and volume modulated arc therapy (VMAT). The choice of technique will depend on the need to protect the organs at risk for increased toxicity with radiation treatment based on the planning CT scan. The planning CT scan will image the lower abdomen and pelvis, from first lumbar vertebra to 3 cm below the buttocks soft tissue projection, with 2.5-mm-thick slices. For both the planning CT scan and treatment, the patient will be in a supine position, with head and knee support and hands resting on the thorax with the bladder comfortably full [41]. Volume delineation and dose prescription will be done according to the guidelines set forth by International Commission on Radiation Units (ICRU) Reports 50 and 62 for 3D-RT and ICRU Report 83 for IMRT or VMAT. The primary tumor and regional lymph nodes will be included in the treatment volumes [42–44]. The following volumes will be considered: gross tumor volume (GTV), which includes the rectal tumor and suspicious lymph nodes; clinical tumor volume 45 Gy (CTV45), which includes the rectal tumor and the regional lymph nodes (delimited superiorly by the common iliac arteries bifurcation and inferiorly by the obturator canal); the planned target volume 45 Gy (PTV45), which includes the CTV45 with a 1-cm external margin except when that margin is the skin; the clinical tumor volume 50.4 Gy (CTV50.4), which includes the GTV and the entire mesorectum and presacral region at involved levels and a 2-cm cephalad and caudal margin beyond any suspicious lymph node, and for tumors located at the anorectal junction, the CTV50.4 will include the GTV and a 1-cm anterolateral and posterior margin in addition to the above mentioned cephalad and caudal margin; and the planned target volume 50.4 Gy (PTV50.4), which includes the CTV50.4 with a 1-cm margin, except at the skin.

During treatment planning the following organs will be considered at risk of unacceptable toxicity during pelvic radiation: bladder, small intestine, and femurs (head of femur). To protect against this risk, during treatment planning, dose-volume histograms for these organs will be independently calculated to ensure that the dose constraints set forth in Table 3 for each of the organs at risk are abided by [45–47].

**Table 3:** Dose-volume constraints

Organ at risk	Dose restriction
Bladder	V40 < 50 %
	V45 < 30 %
Small intestine	No more than 180 cc above 35 Gy
	No more than 100 cc above 40 Gy
	No more than 65 cc above 45 Gy
Femoral head	V25 < 45 %
	V40 < 40 %
	V50 < 10 %

#### Surgery

For all included patients, surgical treatment will be performed 7 to 8 weeks after the end of radiation therapy. The choice of surgical technique will depend on preoperative assessment by a senior surgeon trained in rectal cancer surgery. Total mesorectum excision will be recommended.

#### Management of trial interventions

During treatment, all patients will be observed weekly to assess toxicity. Patients are allowed to receive full supportive care therapies concomitantly during the study. Summary recommendations for dose modification due to treatment emergent adverse events are defined and outlined in Table 4. No individual lapatinib dose reduction or dose escalation will be allowed to manage treatment-related adverse events. In the event of excess toxicity at a dose level of 1250 mg/d of lapatinib, the trial will be stopped and restarted with lapatinib dosed at 1000 mg/d. If a patient requires holding of radiation treatment for 2 or more weeks to manage any adverse event, radiation treatment will be discontinued. In case of radiation treatment interruption, the total dose will be adjusted according to a biological equivalent dose calculation [48]. Any of the study interventions (lapatinib, capecitabine, and radiation therapy) may be discontinued if a patient experiences treatment emergent adverse events judged by the investigator to outweigh its benefits. Treatment with the study drug may be discontinued for any of the following reasons: protocol violation, study termination by the sponsor, loss to follow-up, or death. Additionally patients may discontinue therapy at any time.

**Table 4:** Summary of dose modification guidelines

Adverse event (CTCAE v4.0)	Lapatinib	Capecitabine	Radiation
Hematology: neutrophils or platelets			
Grade –2:	Continue	Hold 7 days^a^	Continue
Grade ≥3:		Hold 7 days^a^	Hold^b^
Dermatology: rash			
Grade –3:	Hold 7 days^a^	Not applicable	
Dermatology: irradiated skin			
Grade ≥3:	Not applicable		Hold^b^
Dermatology: palmar-plantar erythrodysesthesia			
Grade ≥2:	Not applicable	Hold 7 days^a^	Not applicable
Gastroenteroloy: diarrhea			
Grade ≥3:	Discontinue	Hold^a^	Hold^b^
Respiratory: pneumonitis			
Grade ≥3:	Discontinue		
Cardiac dysfunction:			
Asymptomatic LVEF decrease >20 % from baseline and absolute value below the LLN	Discontinue	Not applicable	
Grade –3			
Hepatotoxicity:			
ALT > 3 × ULN AND ALT < 5 × ULN	Continue	Not applicable	
ALT > 3 × ULN AND ALT > 5 × ULN for ≥2 weeks	Discontinue		
ALT > 3 × ULN AND ALT < 5 × ULN for > 4 weeks			
ALT > 3 × ULN AND bilirubin > 2 × ULN (>35 % direct)			
ALT > 3 × ULN AND ALT > 8 × ULN			
ALT > 3 x ULN AND signs and symptoms of hepatitis or hypersensitivity			

^a^Rechallenge if grade 0–1

^b^Restart if grade 0–2

Patients who discontinue any of the study drugs prior to surgical treatment may remain in the study and undergo radiation therapy (if this was not the cause of study drug discontinuation) and surgical treatment as per protocol. Patients who discontinue study drug due to a discontinuation of radiation therapy and are deemed surgically eligible will be surgically treated as per local standard of care. Patients who discontinue study drug will not be replaced.

#### Follow-up

The end of treatment visit will be performed approximately 14 days after the last dose of radiochemotherapy. The end of study visit will be performed 30 days after the surgical intervention. In the event a patient develops any study-related toxicity, he will be followed until its resolution or clinical stabilization.

#### Tumor specimen management

Biopsy fragments will be immediately placed in buffered formalin and allowed to be fixed for 24–48 h. Then, the specimen will be processed overnight for paraffin inclusion. Four-micrometer-thick sections will be obtained from each block, at four levels, and stained with hematoxylin and eosin for histopathological assessment.

The surgical specimen will be delivered to the pathologist fresh and unopened immediately after resection. After inspection and recording of any area of perforation, the non-peritonealized resection margin should be carefully inked. The specimen should then be opened from its anterior surface, excepting the area of the tumor, which should be left unopened to allow for adequate documentation of the circumferential radial margin (CRM). A foam or absorbent paper will then be passed through the residual lumen at the tumor site to aid the penetration of the fixative. The specimen will be allowed to fix in buffered formalin for 24–48 h before further dissection, description, and sampling. The specimen will be sliced transversely at 3–4 mm intervals within a range of 3 cm from the tumor, both proximally and distally. At a minimum, tissue sampling should include four blocks of the tumor, disclosing the deepest level of invasion, involvement of serosal surface or any adjacent organ, and extramural vein invasion; a block from the closest distance of the tumor or extramural deposit or tumor in a lymph node to the non-peritonealized resection margin; a block from the proximal and the distal margins (one each); a block from the transition between the tumor and adjacent normal mucosa; a block from normal mucosa; all lymph nodes identified; any other macroscopic abnormality. If no definite residual tumor can be recognized, the whole scarred area will be blocked for histopathological evaluation. Assessment of pathologic response will be done according to EURECA-CC2 guidelines [49].

#### RAS mutational analysis

Hematoxylin and eosin stained slides will be carefully reviewed by a pathologist to delimit areas with >50 % tumor cells, and adjacent tissue sections will be obtained from formalin-fixed paraffin-embedded tissue for DNA extraction. Specific primers will be used to amplify a 92-bp amplicon of KRAS (exon 2, 3, or 4) or NRAS (exon 2, 3, or 4), which will then be analyzed by high resolution melting (HRM) to screen for mutations. In cases positive by HRM, KRAS exons 2 (codons 12 and 13), 3 (codons 59 and 61), and 4 (codons 117 and 146) and NRAS exons 2 (codons 12 and 13), 3 (codons 59 and 61), and 4 (codons 117 and 146) will be identified by SNaPshot, which is based on the dideoxy single-base extension of an unlabeled oligonucleotide primer. Fragment analyses will be done by capillary electrophoresis. Only patients without KRAS (exon 2, 3, or 4) or NRAS (exon 2, 3, or 4) mutations will be enrolled.

#### Biomarker assessment

EGFR gene amplification and HER-2 gene amplification will be analyzed by fluorescence in situ hybridization on 4-μm thick sections of a representative formalin-fixed paraffin-embedded tissue block. Commercial probes will be used: a test probe that targets EGFR and a control probe for chromosome 7 centromere (CEP7) (Vysis), and a test probe that targets HER-2 and a control probe for chromosome 17 centromere (CEP17) (QBiogene). Slides and probes will be codenatured, and hybridization will take place overnight. After post-hybridization washes, slides will be counterstained with DAPI. Fluorescent images will be sequentially captured with a Cohu 4900 CCD camera, using an automated filter wheel coupled to a Zeiss Axioplan fluorescence microscope and a CytoVision system. Gene amplification will be scored when a ratio EGFR/CEP7 or HER-2/CEP17 is greater than or equal to 2.0 in a minimum of 60 cancer cell nuclei.

EGFR, protein kinase (AKT), and mitogen-activated protein kinase (MAPK) phosphorylation status and Ki-67 will be assessed by immunohistochemical analysis of paraffin-embedded, formalin-fixed tumor tissue. Four-μm sections will be cut and placed in silanized slides. Immunostaining will be performed using a polymer method (BrightVision Poly-HRP-Anti Ms/Rb/Rt IgG, ImmunoLogic, Duiven, The Netherlands). After dewaxing the sections, endogenous peroxidase activity will be inhibited with freshly prepared 0.5 % hydrogen peroxide in distilled water for 20 min. Antigen retrieval will be performed with EDTA buffer, pH 8, for 40 minutes. Incubation with primary antibodies for phosphorylated EGFR (clone Tyr1173 e 53A5), phosphorylated AKT (clone Ser473 e D9E), phosphorylated MAPK (clone Erk1/2,Thr202 e Tyr204), and Ki-67 (clone MIB-1, Dako, Glostrup, Denmark) will be performed overnight at 4 °C, at dilution 1:250, in 1 % bovine serum albumin (BSA) in phosphate-buffered saline (PBS). All incubations will be performed in a humidified chamber. Sections will be developed with a peroxidase substrate solution (0.05 % 3,3-diaminobenzidine tetrahydrochloride, 0.01 % H_2_O_2_ in PBS), counterstained with hematoxylin, dehydrated, and mounted. Appropriate positive and negative controls will be used. Assessment of immunoexpression will be performed by light microscopy at × 400 magnification by a senior pathologist.

### Statistical considerations and quantitative analyses

#### Study endpoints

The primary endpoint is pathologic complete remission (pCR). Pathologic complete remission will be determined on the surgical specimen and defined according to the guidelines recommended by the EURECA-CC2 consensus [2] by a pathologist blinded to the treatment arm.

The secondary endpoint will be the safety profile of the combination lapatinib-capecitabine and radiation therapy in the preoperative setting of patients with resectable rectal cancer assessed by the identification of adverse events and serious adverse events coded with the National Cancer Institute’s Common Terminology Criteria for Adverse Events (CTCAE), version 4.0. An adverse event is defined as any undesirable event associated with the use of a drug, whether or not considered drug related, and includes any side effect, injury, toxicity, or sensitivity reactions, or any undesirable clinical or laboratory event not normally observed in the patient. The following definition of serious adverse event is used: any adverse event that results in death, or is life-threatening, or results in persistent or significant disability or incapacity, requires or prolongs hospitalization, or results in a congenital abnormality or birth defect, or results in any other medically important condition, or an adverse event as a result of an overdose, or is a drug overdose (defined as ≥25 % increase in dose over the protocol-specified dose or more doses are given in a cycle).

Additionally the following exploratory endpoints will be addressed: frequency of and predictive value for the primary endpoint of EGFR amplification status, HER-2/neu amplification status, and predictive value for the primary endpoint of inhibition of EGFR and EGFR downstream proteins on the 14th day of treatment in patients allocated to arm A (MAPK, AKT).

#### Determination of sample size

The trial’s objective is to show the superiority of the lapatinib and capecitabine combination (arm A) over the standard capecitabine (arm B). Thus, the null hypothesis is defined as H_0_: pB ≥ pA, where pA and pB are the probabilities of a pCR for arms A and B, respectively.

The expected sample size calculations were conducted in the context of a Bayesian adaptive randomization design, with stopping rules for early evidence of efficacy/futility and evidence of toxicity. These calculations are based on simulated trial results, which require presetting maximum and minimum total sample size, minimum number of patients fairly randomized to each arm, minimum probability of randomization for each arm, prior distributions for the treatment effect, and stopping rules for efficacy/futility.

We chose a maximum of 80 patients, based on the feasibility of patient’s accrual during the proposed study period. A minimum of 30 patients should be enrolled in the study before stopping the trial due to early evidence of efficacy/inefficacy. However, accrual may stop before the enrollment of 30 patients if there is evidence of toxicity, as defined below. The first 20 patients enrolled in the study will be fairly (non-adaptively) randomized to both arms. During the adaptive randomization phase, the minimum probability of randomization for each arm is set at 0.1.

**Fig. 1:**
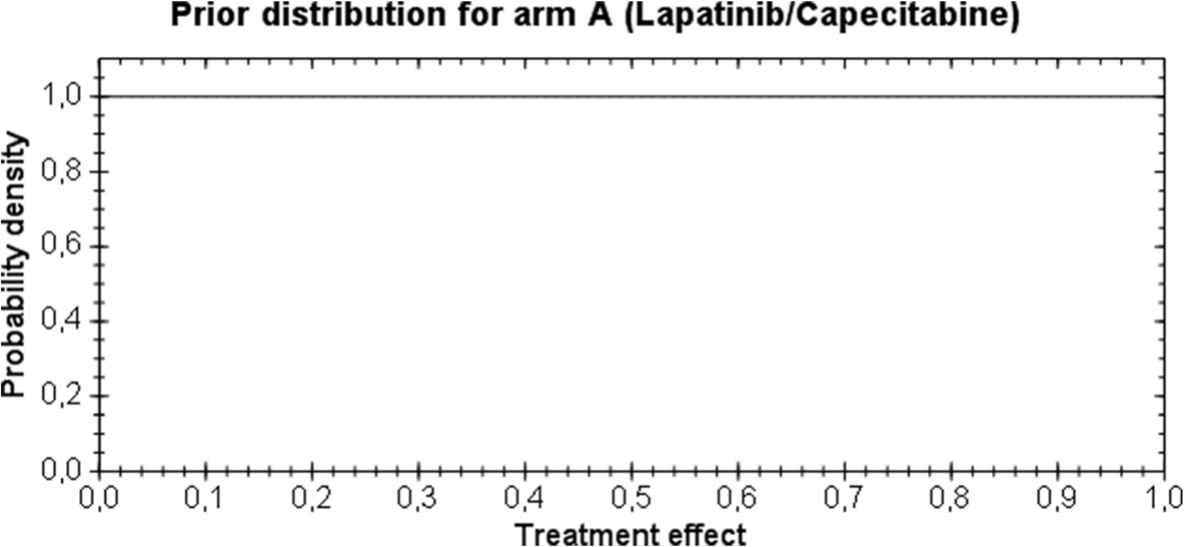
Prior distribution for arm A

**Fig. 2:**
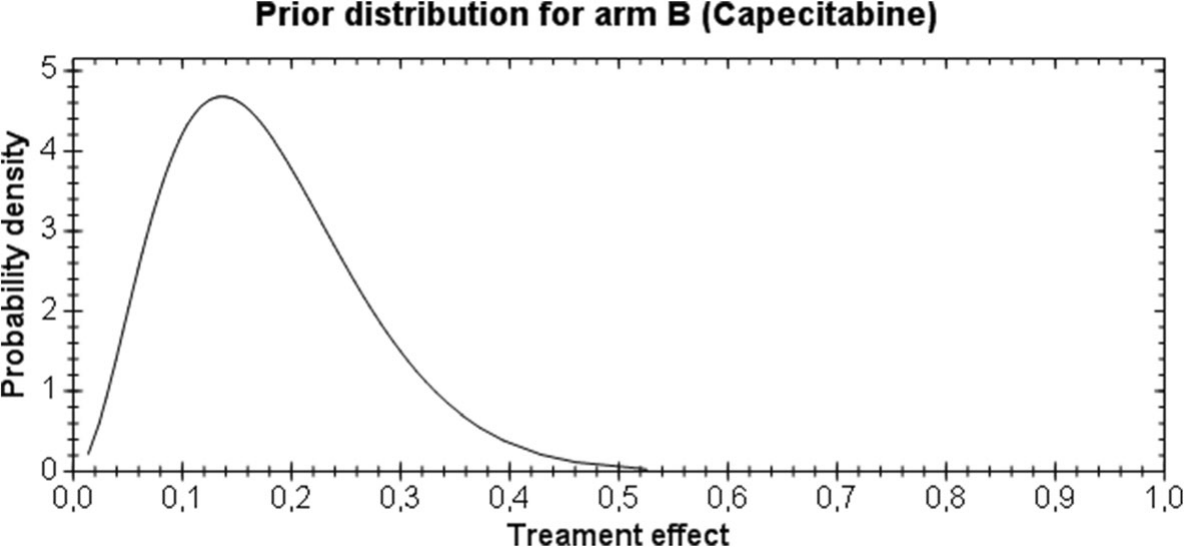
Prior distribution for arm B

For arm A, a non-informative prior for treatment effect, i.e., pA ~ Uniform (0,1), and for arm B, a moderate informative prior for treatment effect pB ~ Beta (1.2, 8.8) are used, as shown in Figs. 1 and 2, respectively.

The trial will be stopped for efficacy if the probability of one arm being superior to the other is greater than 0.95 and for futility if the same probability is less than 0.05. Additionally, the trial stops for futility if the probability of arm A having an effect greater than 0.2 is less than 0.05.

Based on the previous setup, expected sample sizes were computed for three different scenarios, based on 10,000 simulations of trial results:

I. pA = 0.30 and pB = 0.12
II. pA = 0.25 and pB = 0.12
III. pA = 0.12 and pB = 0.12

For scenario I, the expected sample size is 50 with 34 patients enrolled in arm A and 16 in arm B. The probability of choosing arm A as superior is 82 % (equivalent to the power concept in the frequentist framework), and the probability of early stop due to efficacy is 78 %. The expected trial length is 13 months. For scenario II, 56 patients are expected to enroll in the trial with 37 patients in arm A and 29 in arm B. The probability of identifying arm A as superior is 63 % (equivalent to the power concept in the frequentist framework), and the probability of early stop due to efficacy is 64 %. The expected trial length is 15 months. Finally, if no differences exist between the two arms (scenario III), the expected sample size is 60 (30 each arm). The probability of choosing one of the arms as superior is 12 % (equivalent to the probability of type I error in the frequentist framework). The expected trial length is 16 months.

#### Randomization and stratification

The first 20 patients will be enrolled to arm A or B based on a pretrial 1:1 random allocation list generated by the trial statistician and made available to the research team in sealed envelopes. After the first 20 patients, the statistician will provide the randomization result for each patient according to the adaptive design. If a patient discontinues participating in the study, his/her randomization code will not be reused, and the patient will not be allowed to re-enter the study.

#### Quantitative analysis

The following patient populations will be considered for analysis:

- Intention-to-treat (ITT) population: the ITT population is defined as all patients who are randomized. Patients in this population will be analyzed according to the treatment they were randomized to receive, regardless of any errors of dosing.
- Safety population: the safety population will be assessed per treatment arm. For arm A it includes all patients who have completed at least one administration of lapatinib, irrespective of starting treatment with capecitabine (safety population in arm A). For arm B it includes all patients allocated to arm B and who completed at least one administration of capecitabine (safety population in arm B).
- Per protocol (PP) population: the PP population includes all patients who started the allocated treatment and have been submitted to surgical treatment.
- Exploratory subgroup analysis (ESA) population: the ESA population includes patients with complete data for the exploratory biomarkers who did not have any major protocol violation.

All available efficacy and safety data will be included in data listings and tabulations. However, for primary endpoint analysis any missing information regarding the endpoint (loss to follow-up or death prior to surgery and refusal to undergo surgery) will be assumed as absence of pathologic complete response. The impact of this imputation will be tested with a sensitivity analysis. For assessment of the stopping rule of toxicity, loss to follow-up (after all efforts made to assess the reason for it have failed) will be considered to be due to a serious adverse event.

Demographic and baseline characteristics including gender, age, weight, height, clinical stage (American Joint Committee on Cancer’s 7th edition cTN status [40]), EGFR and HER-2 amplification status, and other parameters, as appropriate, will be summarized by treatment groups using descriptive statistics.

The proportion of pCR of each arm will be described and the efficacy of one arm over the other estimated by the posterior probability of superiority and its 95 % credible interval. The trial will be stopped for efficacy if the posterior probability of one arm being superior to the other is greater than 0.95 and for futility if the same probability is less than 0.05. Additionally, the trial stops for futility if the probability of arm A having an effect greater than 0.2 is less than 0.05. The posterior probability of superiority of arm A over arm B will be continuously calculated after the 30th patient outcome is known.

**Fig. 3:**
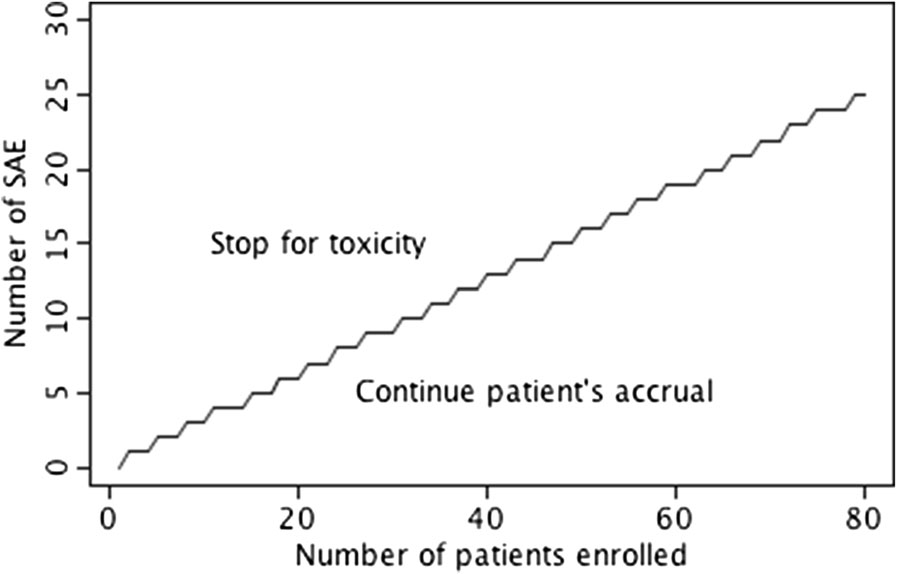
Boundary for early stop due to toxicity

Adverse events will be described as absolute number of patients presenting any given adverse event during treatment, its proportion, and respective 95 % credible intervals. Data will also be summarized as total number of patients presenting at least one serious adverse event and its proportion. For analysis of serious adverse events, a summary posterior probability of a serious adverse event and its 95 % credible interval will be calculated. Patient accrual for arm A will be stopped for toxicity if there is evidence of a serious adverse event posterior probability (SAEPP) greater than 25 %. A conservative threshold of 0.5 will be used for the posterior probability of toxicity, meaning that the trial stops with moderate evidence (50 %) of SAEPP >25 % for arm A. A non-informative prior (Beta (1, 1)) for the proportion of toxic events was chosen. The boundary at each step is plotted in Fig. 3. Should this toxicity boundary be identified for arm A, accrual to the trial is interrupted for excess toxicity at a lapatinib dose level of 1250 mg/d. The trial will then restart with lapatinib at a dose level of 1000 mg/d in arm A, and the data from patients previously randomized to arm B will be used to adjust the prior distribution of pB necessary for random treatment allocation in the Bayesian framework previously defined. In this latter setting, should the toxicity boundary be crossed, accrual to the trial is stopped for toxicity and the combination of lapatinib with capecitabine as radiosensitizer will be considered too toxic for further study.

Biomarkers will be described as the proportion of patients with the given biomarker present in the respective arm and their 95 % credible intervals. Identification of predictive biomarkers will be performed through logistic regression analysis. The model will test the association between activation of EGFR pathway at baseline and pCR (between group analyses) and the discriminatory ability of its inhibition on pCR on arm A (within group analysis). The following biomarkers will be included in the model: EGFR amplification; HER-2/neu amplification; MAPK phosphorylation status; AKT phosphorylation status; EGFR phosphorylation status at baseline (between group analysis) and at baseline and on day 14 biopsy specimens for patients in arm A (within group analysis). Odds ratios will be presented with their 95 % credible intervals, and the c-statistics (area under the receiver operating characteristic curve) will be computed.

### Ethical considerations

The protocol has been written, and the study will be conducted according to the ICH Harmonized Tripartite Guideline on Good Clinical Practice. All patients will be informed about the aims of the study, trial procedures, possible hazards and adverse events, the mechanism of treatment allocation, and procedures to ensure patient data confidentiality before enrollment.

The trial was submitted for ethical review by Portugal’s National Ethics Committee for Clinical Research and was approved on 7 February 2014, deliberation number 2014-RP-02-11.

During the trial, if a protocol amendment is deemed necessary and may significantly affect the safety of patients, the scope of investigation, or the scientific quality of the study, a prior favorable opinion from Portugal’s National Ethics Committee for Clinical Research and approval from the Portuguese National Authority of Medicines and Health Products will be sought before implementation. If a protocol amendment affects only administrative aspects of the study, both the Portuguese National Ethics Committee for Clinical Research and Portuguese National Authority of Medicines will be notified of such administrative changes, but prior approval will not be procured.

Clinical trial insurance has been taken out according to Portuguese law by the sponsor.

## Discussion

To our knowledge, this is the first trial to assess the efficacy of dual inhibition of EGFR and HER-2 as a radiosensitizer in resectable rectal cancer. The trial is designed to include an enriched population of patients with KRAS and NRAS wild-type rectal cancer due to evidence of lack of benefit from anti-EGFR strategies in KRAS or NRAS mutated colorectal tumors. However, the role of KRAS or NRAS mutations in localized rectal cancer is uncertain. There is no information on whether this subgroup of rectal cancer patients have a different prognosis compared to patients with activating mutations nor if absence of mutation in KRAS or NRAS is predictive of response to standard radiochemotherapy. Thus, a randomized trial with an active comparator was needed to answer this question. The choice of a randomized adaptive Bayesian design allows an optimized sample size as well as a concurrent comparator. This design optimizes the trial’s sample size and protects eligible patients from randomization to a weakly performing arm, thus improving our ability to decide whether further study of lapatinib in this setting is warranted [50, 51].

The primary objective of the trial is to show that lapatinib-capecitabine radiochemotherapy is superior to capecitabine radiochemotherapy. The choice of complete pathologic response as the primary outcome measure allows such interpretation since it is a feasible and reproducible short-term endpoint and is reported to be associated with improved cancer-specific survival [7]. The choice to use full doses for each treatment component maximizes the probability of treatment response. As with any novel treatment combination, tolerability will be an important issue. The experimental arm has never been studied in this setting. Therefore, we have integrated into the Bayesian design a specific stopping rule to address this. This rule was designed to reproduce the performance of classic phase I trials, which are designed to explore the maximum tolerated dose of a given agent or combination while protecting as many patients as possible from excessive risks. The main anticipated toxicity is diarrhea; therefore, mandatory pre-emptive antidiarrheal medication will be recommended for every patient. Should excess toxicity be an issue with a standard dose of lapatinib, we have stipulated a reset of the trial with a lower dose of lapatinib. However, this should not impact the assessment of clinical value of this combination, as the lower dose level of lapatinib is still associated with a C_min_ above that which is required for antitumor effect. Additionally, the incorporation of a lead-in period of lapatinib together with tumor sampling before and after the 2-week lead-in period will provide further information on the pharmacodynamics of lapatinib and direct evidence of on-target effect.

This trial includes an exploratory biomarker program. This was designed to better understand the role that EGFR inhibitors and lapatinib in particular play in this setting and to identify potentially predictive biomarkers for response.

In conclusion, the LaRRC trial will inform on the role of dual inhibition of EGFR and HER-2 in the neoadjuvant treatment of resectable rectal cancer. The choice of a Bayesian adaptive randomization together with an initial lead-in treatment phase with lapatinib in the experimental arm provides the best chance to maximize the information collected while effectively balancing the risks and potential benefits for eligible patients.

## Trial status

The trial is currently recruiting.

## Abbreviations

3D-RT: 3D conformal external beam radiation
CRM: Circumferential radial margin
CTV45: Clinical tumor volume 45 Gy
CTV50.4: Clinical tumor volume 50.4 Gy
CTCAE: Common Terminology Criteria for Adverse Events
DPD: Dihydropyrimidine dehydrogenase
EGFR: Epidermal growth factor receptor
ESA: Exploratory subgroup analysis
GTV: Gross tumor volume
HRM: High resolution melting
IMRT: Intensity modulated radiotherapy
ITT: Intention to treat
pCR: Pathologic complete remission
PP: Per protocol
PTV45: Planned target volume 45 Gy
PTV50.4: Planned target volume 50.4 Gy
SAEPP: Serious adverse event posterior probability
ULN: Upper limit of normality
VMAT: Volume modulated arc therapy
